# Comparative analysis of cerebrospinal fluid metabolites in Alzheimer’s disease and idiopathic normal pressure hydrocephalus in a Japanese cohort

**DOI:** 10.1186/s40364-018-0119-x

**Published:** 2018-01-22

**Authors:** Yuki Nagata, Akiyoshi Hirayama, Satsuki Ikeda, Aoi Shirahata, Futaba Shoji, Midori Maruyama, Mitsunori Kayano, Masahiko Bundo, Kotaro Hattori, Sumiko Yoshida, Yu-ichi Goto, Katsuya Urakami, Tomoyoshi Soga, Kouichi Ozaki, Shumpei Niida

**Affiliations:** 1 Medical Genome Center, National Center for Geriatrics and Gerontology, 7-430 Morioka-cho, Obu, Aichi 474-8511 Japan; 2 Institute for Advanced Biosciences, Keio University, 246-2 Mizukami, Kakuganji, Tsuruoka, Yamagata 997-0052 Japan; 3 Research Center for Global Agromedicine, Obihiro University of Agriculture and Veterinary Medicine, 2-11 Inada-cho, Obihiro, Hokkaido 080-8555 Japan; 4 Department of Experimental Neuroimaging, National Center for Geriatrics and Gerontology, Obu, Aichi 474-8511 Japan; 5 Medical Genome Center, National Center of Neurology and Psychiatry, Kodaira, Tokyo, 187-8551 Japan; 6 Department of Biological Regulation, School of Health Science, Faculty of Medicine, Tottori University, Yonago, Tottori 683-8503 Japan

**Keywords:** Alzheimer’s disease, Idiopathic normal pressure hydrocephalus, Diagnostic marker, Cerebrospinal fluid, Serine, Glycerate, N-acetylneuraminate, 2-hydroxybutyrate

## Abstract

**Background:**

Alzheimer’s disease (AD) is a most common dementia in elderly people. Since AD symptoms resemble those of other neurodegenerative diseases, including idiopathic normal pressure hydrocephalus (iNPH), it is difficult to distinguish AD from iNPH for a precise and early diagnosis. iNPH is caused by the accumulation of cerebrospinal fluid (CSF) and involves gait disturbance, urinary incontinence, and dementia. iNPH is treatable with shunt operation which removes accumulated CSF from the brain ventricles.

**Methods:**

We performed metabolomic analysis in the CSF of patients with AD and iNPH with capillary electrophoresis-mass spectrometry. We assessed metabolites to discriminate between AD and iNPH with Welch’s *t*-test, receiver operating characteristic (ROC) curve analysis, and multiple logistic regression analysis.

**Results:**

We found significant increased levels of glycerate and N-acetylneuraminate and significant decreased levels of serine and 2-hydroxybutyrate in the CSF of patients with AD compared to the CSF of patients with iNPH. The ROC curve analysis with these four metabolites showed that the area under the ROC curve was 0.90, indicating good discrimination between AD and iNPH.

**Conclusions:**

This study identified four metabolites that could possibly discriminate between AD and iNPH, which previous research has shown are closely related to the risk factors, pathogenesis, and symptoms of AD. Analyzing pathway-specific metabolites in the CSF of patients with AD may further elucidate the mechanism and pathogenesis of AD.

**Electronic supplementary material:**

The online version of this article (10.1186/s40364-018-0119-x) contains supplementary material, which is available to authorized users.

## Background

Alzheimer’s disease (AD) is the most common type of dementia in the world, which concerns approximately 60–70% cases of dementia, and it is becoming a significant social issue because of the growing aging population.

In general, AD is diagnosed based on the presence of cognitive impairment and by neuropsychological testing according to the National Institute of Neurological and Communicative Disorders and Stroke and the Alzheimer’s Disease and Related Disorders Association criteria [1]. Currently, there is no curative or radical treatment for AD, although some medicines have been developed to reduce the symptoms [2]. However, some symptoms of AD are similar to those of other neurodegenerative diseases, such as idiopathic normal pressure hydrocephalus (iNPH). iNPH is caused by the accumulation of cerebrospinal fluid (CSF) in the brain and causes gait disturbance, urinary incontinence, and dementia. In contrast to other dementias, iNPH is treatable by shunt operation that removes the accumulated CSF [3]. Therefore, a precise diagnosis that discriminates patients with AD from patients with iNPH is essential for proper treatment at the early stages of these diseases. Presently, increased phosphate tau (p-tau) and decreased amyloid-beta 1-42 (Aβ42) in the CSF are used as established AD diagnostic markers [4]. However, p-tau accumulates after synaptic degeneration and Aβ42 is difficult to accurately quantify. Therefore, we tried to find additional biomarkers, capable of precisely detecting AD before neurodegeneration progresses.

Recently, accumulated evidence has indicated that AD is a type of metabolic disease in the brain [5, 6]. The brain is the most energy-consuming organ and glucose is an essential and dominant energy source for the brain [7]. Progressive regional cerebral glucose metabolism reduction, correlated with the symptom severity, has been found in the brain of patients with AD [8, 9]. Mills et al. (2013) performed RNA-Seq analysis in the parietal cortex of patients with AD and reported that two enzymes, ACOT1 and ACOT2*,* which are involved in lipid metabolism, were upregulated. They also found a downregulation of TERC, which is involved in the synthesis of very long chain fatty acids [10]. In addition, impairment of the insulin response, as is seen in diabetes mellitus and metabolic syndrome, was revealed as a risk factor for AD [11–13]. These reports suggested that the pathomechanism of AD is strongly related to a disturbance in brain energy metabolism and homeostasis. The disturbance could induce metabolite alterations in the body fluids of patients with AD, such as in the plasma, serum, and CSF. Therefore, metabolome analysis of body fluids in AD has been actively performed to identify new diagnostic markers. Especially, the CSF is thought to be a superior analyte than other body fluids because it is in direct contact with the extracellular space of the brain and thus directly reflects the biological changes in the pathological brain processes in AD.

So far, several pertinent reports have been published and several diagnostic markers for AD have been suggested. For example, D’ Aniello et al. (2005) conducted high performance liquid chromatography (HPLC) analysis and found L-glutamine was increased and L-asparate was decreased in the CSF of patients with AD than in the CSF of controls [14]. Czech et al. (2012) reported that increased cysteine with decreased uridine was the optimal combination to identify mild AD, and increased cortisol levels were associated with the progression of AD in a European cohort [15]. Ibanez et al. (2012) performed capillary electrophoresis-mass spectrometry (CE-MS) to investigate metabolome changes in the CSF of patients at different AD stages and found that choline, dimethylarginine, arginine, valine, proline, serine, histidine, creatine, carnitine, and suberylglycine could be possible disease progression markers [16]. Furthermore, they conducted ultra-high-performance liquid chromatography-time-of-flight mass spectrometry (TOFMS) and found uracil and uridine were good candidate biomarkers for AD, as has been previously described [15, 17].

However, different metabolomic platforms and methods detect different metabolites, thus utilizing multiple types of metabolomic platforms would provide a wider perspective of metabolomics information under study. In addition, differences in ethnicity, culture, and education may influence decision making regarding the diagnosis, symptoms, and severity of AD [18]. Accordingly, accumulating information related to AD pathology from differential ethnoracial cohorts and with differential methods is essential.

From these points of view, we performed metabolomic analysis of the CSF of patients with AD or iNPH in Japanese cohorts with capillary electrophoresis-TOFMS (CE-TOFMS). Our present findings would support the utility of metabolomics analysis for discriminate between AD and iNPH.

## Methods

### Subjects

The characteristics of the study patients with AD and iNPH are summarized in Table 1. AD and iNPH were diagnosed according to previously published criteria [1, 19]. Bioresources were largely obtained from the biobank of the National Center for Geriatrics and Gerontology (NCGG, Aichi, Japan) and partly donated by the National Center of Neurology and Psychiatry (NCNP, Tokyo, Japan) and the Department of Biological Regulation, School of Medicine, Tottori University (Tottori, Japan). All samples were obtained with the written informed consent of the patients before sampling between 2011 and 2015. This study was reviewed and approved by the ethics committees of all participating institutes and biobanks.

**Table 1:** Subject characteristics.

	Metabolomics		Validation	
	AD	iNPH	AD	iNPH
Subject No.	39	19	42	38
Mean age (SD)	73.5 (9.8)	77.8 (4.5)	74.1 (9.6)	77.9 (5.6)
Age range	47-86	70-86	47-86	57-87
P-value	0.03		0.03	
Male/female	14/25	9/10	15/27	22/16
P-value	0.24		0.09	
Mean MMSE (SD)	21.4 (5.1)	23.5 (2.9)	21.2 (5.0)	22.1 (3.1)
P-value	0.08		0.46	
Missing values	6	0	6	0

### Collection of CSF

CSF was collected by lumbar puncture and centrifuged to remove debris. The supernatant was aliquoted into low protein-binding tubes and immediately frozen in liquid nitrogen and stored at −80 °C until use.

### Measurement of p-tau and Aβ42

To detect the concentration of *p*-tau and Aβ42 in the CSF, the enzyme-linked immunosorbent assay systems of INNOTEST β-AMYLOID_(1-42)_ and INNOTEST PHOSPHO-TAU_(181P)_ were used according to the manufacturer’s instructions (Fujibireo Inc., Tokyo, Japan).

### Metabolites extraction from CSF

Frozen CSF samples were thawed and 40 μL aliquots were placed into 360 μL of methanol containing internal standards (20 μmol/L each of methionine sulfone and D-camphor-10-sulfonic acid). The solutions were thoroughly mixed, and then both 400 μL of chloroform and 160 μL of Milli-Q water were added, followed by centrifugation at 10,000 × *g* for 3 min at 4 °C. The aqueous layer was transferred to a 5-kDa-cutoff filter (Human Metabolome Technologies, Tsuruoka, Japan) to remove proteins. The filtrate was dried using a centrifuge concentrator and reconstituted with 50 μL of Milli-Q water containing reference compounds (200 μmol/L each of 3-aminopyrrolidine and trimesic acid) prior to CE-TOFMS analysis.

### Metabolome analysis by CE-TOFMS

All CE-TOFMS experiments were performed using an Agilent 1600 Capillary Electrophoresis system (Agilent technologies, Santa Clara, CA), an Agilent 6220 TOF LC/MS system, an Agilent 1200 series isocratic HPLC pump, a G1603A Agilent CE-MS adapter kit, and a G1607A Agilent CE-electrospray ionization (ESI)-MS sprayer kit. In the anionic metabolites analysis, ESI sprayer was replaced with a platinum needle instead of the initial stainless steel needle [20]. The other conditions relating to the CE-ESI-MS sprayer were identical as received. For CE-MS system control and data acquisition, we used the Agilent MassHunter software.

### Cationic metabolome analysis

For cationic metabolome analysis, a fused-silica capillary (50 μm i.d. × 100 cm) filled with 1 mol/L formic acid as the electrolyte was used [21]. A new capillary was flushed with the electrolyte for 20 min, and the capillary was equilibrated for 4 min by flushing with the electrolyte before each run. Sample solution was injected at 5 kPa for 3 s and a positive voltage of 30 kV was applied. The temperatures of the capillary and sample trays were maintained at 20 °C and 4 °C, respectively. Methanol/water (50% *v*/v) containing 0.1 μmol/L hexakis(2,2-difluoroethoxy)phosphazene was delivered as sheath liquid at 10 L/min. ESI-TOFMS was operated in the positive ion mode, and the capillary voltage was set at 4 kV. The flow rate of heated nitrogen gas (heater temperature, 300 °C) was maintained at 10 psig. In TOFMS, the fragmentor, skimmer, and Oct RF voltages were set at 75, 50, and 125 V, respectively. Automatic recalibration of each acquired spectrum was performed using the masses of reference standards ([^13^C isotopic ion of protonated methanol dimer (2CH_3_OH + H)]^+^, *m/z* 66.06306) and ([hexakis(2,2-difluoroethoxy)phosphazene + H]^+^, *m/z* 622.02896). Exact mass data were acquired at the rate of 1.5 cycles/s over a 50 to 1000 *m/z* range.

### Anionic metabolome analysis

For the anionic metabolome analysis, a COSMO(+) capillary (50 μm i.d. × 105 cm, Nacalai Tesque, Kyoto, Japan) filled with 50 mmol/L ammonium acetate (pH 8.5) as the electrolyte was used [20]. Before the first use, a new capillary was successively flushed with the electrolyte, 50 mmol/L acetic acid (pH 3.4), and then the electrolyte again for 10 min each. Before each run, the capillary was equilibrated by flushing with 50 mmol/L acetic acid (pH 3.4) for 2 min and then with the electrolyte for 5 min. Sample was injected at 5 kPa for 30 s and a negative voltage of 30 kV was applied. The temperatures of the capillary and sample trays were maintained at 20 °C and 4 °C, respectively. Ammonium acetate (5 mmol/L) in 50% (*v*/v) methanol/water solution that contained 0.1 μmol/L hexakis(2,2-difluoroethoxy)phosphazene was delivered as sheath liquid at 10 μL/min. ESI-TOFMS was operated in the negative ion mode, and the capillary voltage was set at 3.5 kV. The flow rate of heated nitrogen gas (heater temperature, 300 °C) was maintained at 10 psig. In TOFMS, the fragmentor, skimmer, and Oct RF voltages were set at 100, 50, and 200 V, respectively. Automatic recalibration of each acquired spectrum was performed using the masses of reference standards ([^13^C isotopic ion of deprotonated acetate dimer (2CH_3_COOH − H)]^−^, *m/z* 120.03841) and ([hexakis(2,2-difluoroethoxy)phosphazene + deprotonated acetate(CH_3_COOH − H)]^−^, *m/z* 680.03554). Exact mass data were acquired at the rate of 1.5 cycles/s over a 50 to 1000 *m/z* range.

### Statistical analyses

Comprehensive metabolic data were processed using our proprietary software (MasterHands) [22–24]. The peaks were identified by matching *m/z* values and normalized migration times of corresponding authentic standard compounds. Statistical analysis was performed using Welch’s *t*-test, receiver operating characteristic (ROC) curve analysis, and Pearson’s correlation analysis in R version 3.3.2 (2016-10-31) [25]. Multiple logistic regression analysis was performed with Statflex ver. 6 (Artech Co., Ltd., Osaka, Japan). A *P*-value <0.05 was considered to be significant.

## Results

First, we performed comprehensive metabolic analysis of the CSF to identify characteristic metabolites in the patients with AD and iNPH with the screening subjects indicated in Table 1 (column: Metabolomics). Eighty-three anionic and 60 cationic metabolites were detected in this analysis (Additional file 1). Among these, 18 metabolites showed a *P*-value <0.05 and an area under the ROC curve (AUC) > 0.7 between AD and iNPH with several missing values (Table 2). Therefore, to validate the concentration of these metabolites in the CSF of patients with AD and iNPH, we repeated CE-TOFMS with additional samples (Table 1, column: Validation). Undecanoate (PubChem ID: 8180) and N-acetylhistidine (PubChem ID: 273,260) were excluded from further analysis as false positives because they are not metabolized in the human brain [26]. Nine metabolites indicated *P*-value <0.05 and an AUC > 0.7 between AD and iNPH without missing values (Table 3). Of these metabolites, glycerate (PubChem ID: 752) and N-acetylneuraminate (Neu5Ac, PubChem ID: 3568) were increased in the CSF of patients with AD than of patients with iNPH, while the other seven metabolites were decreased.

**Table 2:** Statistically significant metabolites between AD and iNPH in the metabolomics

		Mean concentration, μmol/L (SD)				Welch’s t-test	ROC	AD/iNPH	Valid value		Missing value	
PubChem ID	Metabolite	AD		iNPH		*P*-value	AUC	Fold change	AD	iNPH	AD	iNPH
752	Glycerate	60.61	(30.94)	26.23	(6.22)	6.E-07	0.88	2.31	32	16	7	3
1060	Pyruvate	45.22	(13.45)	68.34	(12.95)	1.E-04	0.89	0.66	12	16	27	3
3441	2-Oxoisopentanoate	4.29	(1.19)	5.64	(1.29)	0.001	0.78	0.76	30	19	9	0
3568	N-Acetylneuraminate	16.75	(3.89)	13.35	(3.2)	0.001	0.76	1.25	39	19	0	0
617	Serine	24.63	(3.9)	30.12	(6.15)	0.002	0.77	0.82	39	19	0	0
273,260	N-Acetylhistidine^a^	1.19	(0.88)	0.88	(0.92)	0.002	0.72	1.36	39	19	0	0
8180	Undecanoate^a^	0.54	(0.17)	0.71	(0.09)	0.003	0.81	0.76	17	7	22	12
3,527,278	4-Methyl-2-oxopentanoate	2.64	(0.85)	3.51	(0.98)	0.003	0.74	0.75	39	19	0	0
11,266	2-Hydroxybutyrate	18.08	(5.98)	23.14	(6.06)	0.005	0.74	0.78	39	19	0	0
525	Malate	1.17	(0.33)	1.54	(0.46)	0.005	0.73	0.76	39	19	0	0
602	Alanine	32.03	(8.53)	42.82	(14.42)	0.006	0.77	0.75	39	19	0	0
876	Methionine	2.79	(1.08)	3.67	(1.07)	0.006	0.76	0.76	39	19	0	0
205	Threonine	27.56	(6.43)	34.81	(9.53)	0.006	0.73	0.79	39	19	0	0
232	Arginine	23.08	(4.5)	26.79	(4.6)	0.006	0.71	0.86	39	19	0	0
64,969	3-Methylhistidine	1.12	(0.88)	1.81	(0.92)	0.010	0.79	0.62	39	19	0	0
1081	Citramalate	1.20	(0.96)	0.57	(0.12)	0.010	0.74	2.12	19	8	20	11
1175	Urate	20.84	(11.36)	30.61	(15.74)	0.023	0.73	0.68	39	19	0	0
866	Lysine	30.00	(5.84)	36.35	(10.66)	0.023	0.72	0.82	39	19	0	0

^a^Undecanoate and N-Acetylhistidine were excluded from further analysis

*AD* Alzheimer’s disease, *iNPH* idiopathic normal pressure hydrocephalus, *AUC* area under the curve, *ROC* receiver operator characteristic

**Table 3:** Statistically significant metabolites between AD and iNPH in the validation

	Mean concentration, μmol/L (SD)				Welch’s t-test	ROC	AD/iNPH
Metabolite	AD		iNPH		*P*-value^a^	AUC	Fold change
Serine	29.1	(5.3)	34.5	(4.9)	1.6E-04	0.78	0.84
Glycerate	54.6	(22.2)	37.3	(12.9)	2.0E-04	0.71	1.46
3-Methylhistidine	1.1	(0.9)	2.1	(1.2)	6.4E-04	0.82	0.52
Threonine	29.0	(7.0)	39.4	(12.9)	7.4E-04	0.77	0.74
Methionine	2.6	(1.0)	3.6	(1.2)	9.4E-04	0.77	0.71
Urate	19.9	(8.6)	28.7	(9.9)	1.3E-03	0.78	0.70
N-Acetylneuraminate	16.1	(3.9)	12.9	(3.2)	1.9E-03	0.75	1.25
Alanine	33.0	(8.4)	42.4	(12.6)	3.9E-03	0.73	0.78
2-Hydroxybutyrate	17.5	(4.6)	22.2	(6.7)	0.009	0.72	0.79

^a^Bonferroni correction was applied

*AD* Alzheimer’s disease, *iNPH* idiopathic normal pressure hydrocephalus, *AUC* area under the curve, *ROC* receiver operator characteristic

**Fig. 1:**
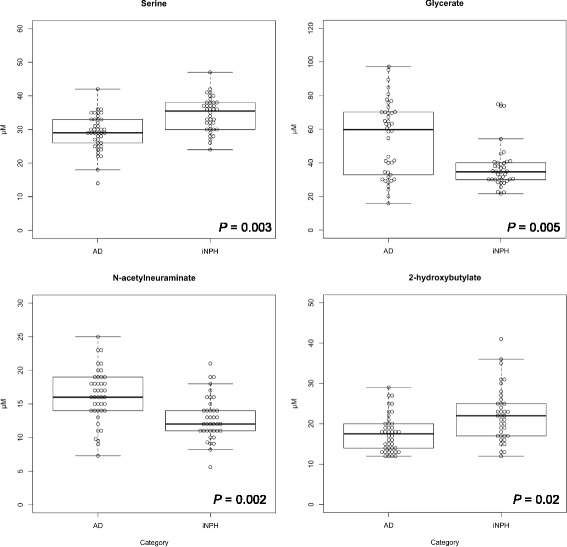
Statistically significant metabolites in the cerebrospinal fluid between Alzheimer’s disease and idiopathic normal pressure hydrocephalus. Four metabolites were statistically significant in the multiple logistic regression analysis

**Fig. 2:**
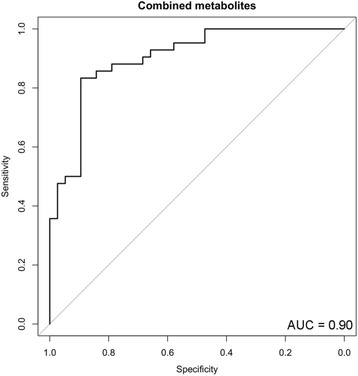
Receiver operator characteristic (ROC) curve analysis of statistically significant metabolites in the cerebrospinal fluid between Alzheimer’s disease (AD) and idiopathic normal pressure hydrocephalus (iNPH). ROC curve analysis was performed to compare the predictive power of AD and iNPH with the combined metabolites

Next, we performed multiple logistic regression analysis with these nine metabolites, setting age as a covariate. Using stepwise regression, we found statistical significance for four metabolites, serine (PubChem ID: 617), glycerate, Neu5Ac, and 2-hydroxybutylate (2-HB, PubChem ID: 11,266) (Fig. 1 and Table 4). Formulation of the regression coefficient of these four metabolites was as follows; (−0.1198) × age + (−0.2508) × serine + (0.05715) × glycerate + (0.37226) × Neu5Ac + (−0.1705) × 2-HB + 12.3001. When the cutoff value was set to 9.73, sensitivity and specificity were highest (Fig. 2, AUC = 0.90, sensitivity = 0.86, specificity = 0.84, and the odds ratio was 32.0).

**Table 4:** Statistically significant metabolites in multiple logistic regression analysis

Metabolite	*P*-value	Odds ratio	CI 95%
Serine	0.002	0.78	0.66-0.91
Glycerate	0.009	1.06	1.01-1.10
N-Acetylneuraminate	0.003	1.45	1.14-1.85
2-Hydroxybutyrate	0.034	0.84	0.72-0.99

Age was used as a covariate

**Fig. 3:**
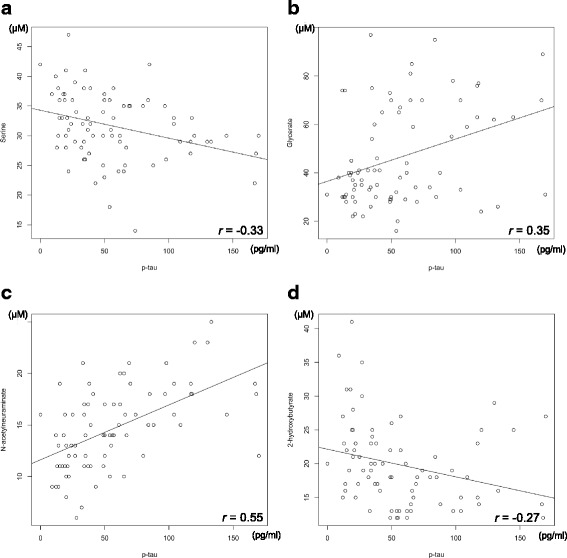
Correlation diagram of p-tau versus the four metabolites. (**a**) serine, (**b**) glycerate, (**c**) Neu5Ac, and (**d**) 2-HB with regression lines are indicated

**Fig. 4:**
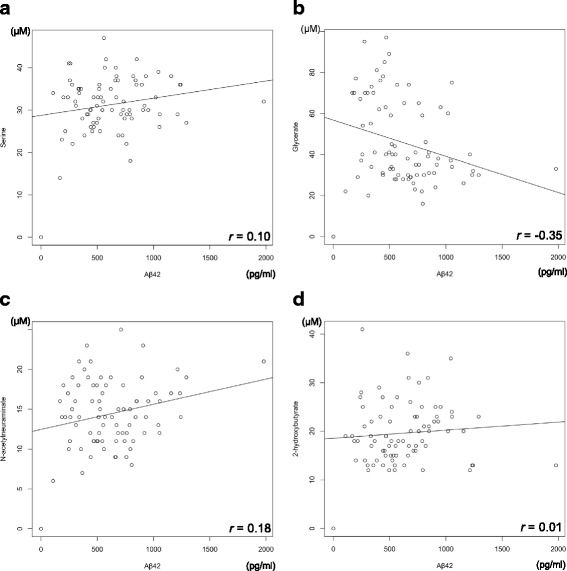
Correlation diagram of Aβ42 versus the four metabolites. (**a**) serine, (**b**) glycerate, (**c**) Neu5Ac, and (**d**) 2-HB with regression lines are indicated

**Fig. 5:**
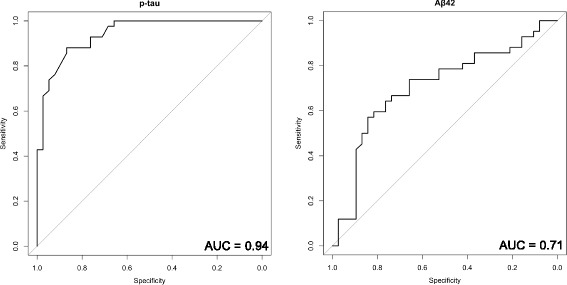
Receiver operator characteristic (ROC) curve analysis of *p*-tau and Aβ42 in the cerebrospinal fluid between Alzheimer’s disease and idiopathic normal pressure hydrocephalus. ROC curve analysis was performed to compare the predictive power of p-tau (left) and Aβ42 (right)

Further, we examined the correlation between *p*-tau, Aβ42, and these four metabolites. Correlation coefficient values were −0.33, 0.35, 0.55, and −0.27 between *p*-tau and serine, glycerate, Neu5Ac, and 2-HB, respectively, showing a weak to moderate correlation between *p*-tau and the four metabolites (Fig. 3). On the other hand, the correlation coefficients between Aβ42 and the four metabolites were 0.10, −0.35, 0.18, and 0.01 for serine, glycerate, Neu5Ac, and 2-HB, respectively, showing weak or absent correlations for the first three metabolites and a negative correlation for glycerate (Fig. 4). When ROC curve analysis was performed between AD and iNPH with p-tau and Aβ42, the AUC values were 0.94 and 0.71 for p-tau and Aβ42, respectively (Fig. 5). These results indicate that these metabolites combined may have a discriminatory power equal to that of *p*-tau.

## Discussion

We found the combination of four metabolites, serine, glycerate, Neu5Ac, and 2-HB could contribute to distinguishing AD from iNPH (Table 4, Fig. 2). We searched the KEGG Pathway Database [27] (http://www.genome.jp/kegg/pathway.html) and found that glycerate is the intermediate metabolite in the pentose phosphate pathway (PPP, KEGG ID: map00030), amino acid metabolism (KEGG ID: map00260), and glycerolipid metabolism (KEGG ID: map00561) in humans.

PPP is one of the glucose metabolic pathways generating pentose and nicotinamide adenine dinucleotide phosphate (NADPH). NADPH has an antioxidant reducing activity and an important role in opposing oxidative stress [28]. In the brain of patients with AD, oxidative stress signatures are observed at the very early stage of the disease [29–31] and PPP is activated to provide NAPDH to counteract oxidative stress [32, 33]. In addition, in the state of hypoxia, which is reportedly a risk factor for AD [34], the PPP preferentially metabolizes glucose instead of the common glycolysis pathway [35], also suggesting the activation of PPP in the brain of patients with AD. Together, increased glycerate in the CSF of patients with AD may reflect the activation of PPP to compensate for the failure in brain functions that accompany AD progression.

Glycerate is also generated in amino acid metabolism, glycine, serine, and threonine. Some reports have indicated alterations in amino acid metabolism in AD [36–38]. Especially, Madeira et al. reported increased levels of D-serine and total serine concentrations in the CSF of patients with AD [39]. They indicated that the amyloid beta oligomer activated serine racemase, an enzyme which converts L-serine to D-serine. In our study, although we did not detect D-serine sole concentration, total serine concentration was decreased in the patients with AD (Fig. 1). However, as we included patients with iNPH as controls, it is difficult to directly compare our results to those of the other studies, which included healthy individuals as controls. However, there is a report indicating phosphatidylserine synthase (PSS) was activated in the aging rat brain [40]. PSS incorporates serine into phosphatidylethanolamine or phosphatidylcholine and generates phosphatidylserine. As aging is a risk factor for AD [40] and phosphatidylserine administration improved several cognitive measures in AD [41], serine reduction in the CSF of patients with AD may reflect the neuroprotective role of PSS. Moreover, serine is converted to hydroxypyruvate by transamination, followed by conversion to glycerate by glycerate dehydrogenase [42]. Additionally, glycerate is generated in the serine degradation pathway [43, 44]. Therefore, increased glycerate and decreased serine in the CSF of patients with AD seem to be justifiable.

Another group indicated that there was no statistically significant difference in serine concentration between the CSF of patients with AD and that of controls [14, 45]. However, these studies were performed with relatively small sample sizes and amino acids concentration in the CSF seemingly influenced by the content of daily diet [46], further studies with larger sample sizes are needed to elucidate the relations between serine concentration in the CSF and AD.

Glycerate is also produced during glycerolipid metabolism. Malaisse et al. indicated that triglyceride species were increased in the brain tissue of Type II diabetic rats [47]. Type II diabetes is known as a risk factor for AD [48, 49] and AD is known as the Type III diabetes [50], suggesting glycerolipid metabolism accelerated in the AD brain and may result in the accumulation of glycerate in the CSF.

Other than glycerate, Neu5Ac was increased in the CSF of AD. Neu5Ac is the most abundant sialic acid in nature and a component of gangliosides [51]. Gangliosides are abundant in neural cell membranes and have important roles in the organization of lipid rafts [52]. Lipid rafts are the subdomains of the plasma membrane that integrate numerous types of lipid proteins having important roles in cell signaling, cell-cell adhesion, and intracellular vesicular trafficking. There are some reports indicating that lipid rafts contain many types of AD associated proteins [53–56] and aberrations in the structure of the lipid rafts are considered to lead to AD [57]. Kracun et al. indicated that there was significant decrease of gangliosides in the brain of patients with AD and in the aging population, suggesting accelerated degradation of gangliosides accompanied by neuronal cell death [58]. Hence, the increased level of Neu5Ac in the CSF of patients with AD may reflect that neuronal and lipid raft destruction may accompany AD progression.

**Fig. 6:**
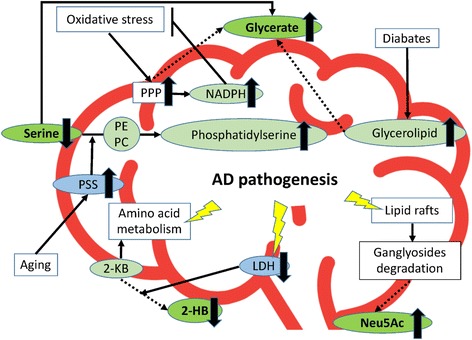
A model of the relationship of Alzheimer’s disease-specific metabolites indicated in this study. Serine, glycerate, Neu5Ac, and 2-HB are depicted with the AD-related metabolites, phenomenon, structures and metabolic pathways. Metabolites detected in this study are indicated in green oval with bold letters. Other related metabolites are indicated in light green oval. Enzymes are indicated in light blue oval. The phenomena, structure and pathways known to participate with AD pathogenesis are indicated in square. Valid allows indicate the interactions previously reported. Dotted allows indicate predicted interactions. Red bold line indicates the brain and CSF boundary. 2-KB: 2-ketobutyrate; 2-HB: 2-hydroxybutyrate; AD: Alzheimer’s disease; LDH: lactate dehydrogenase; NADPH: nicotinamide adenine dinucleotide phosphate; Neu5Ac: N-acetylneuraminate; PC: phosphatidylcholine; PE: phosphatidylethanolamine; PPP: pentose phosphate pathway; PSS: phosphatidylserine synthase

In this study, 2-HB was decreased in the CSF of patients with AD. 2-HB is derived from 2-ketobutyrate (2-KB) dehydration by lactate dehydrogenase (LDH) [59]. 2-KB is an important intermediate metabolite of amino acid metabolism, which is reportedly altered in AD [36–38]. In addition, in the brain of AD model mice, LDH expression was decreased [60], which may result in decreased 2-HB production. Hence, the four metabolites detected in this study are likely related to AD risk factors, pathogenesis, and/or symptoms (Fig. 6). Also, all these metabolites are found at the first time as the AD related ones in the CSF.

The metabolites described here likely participate in several pathways; however, we could not find other statistically significant metabolites included in these pathways. Easily detectable metabolites differ from metabolomic platforms and methods; therefore, other metabolites participating in the pathways indicated here could not be detected in this study. Further analyses with several metabolomic platforms are needed to complement metabolomic information for AD.

Recently, fluorodeoxyglucose (FDG)-positron emission tomography (PET), which detects the cerebral metabolic rates of glucose, has been used to diagnose AD [61], indicating the usefulness of measuring metabolic pathways for diagnosing AD. Other than glucose metabolic rates, several studies have indicated numerous types of metabolic pathways to be influenced relatively early in AD progression [6, 12, 62]. However, it is still difficult to make a precise and early diagnosis for AD at present. To establish the methods of precise and early diagnosis, comprehensive analyses for the pathway specific metabolites altered in AD would be effective.

In this study, the concentration of the four metabolites and established AD biomarker, p-tau, in the CSF were weakly correlated, although another established AD biomarker, Aβ42, did not show similarly significant results (Figs. 3, 4). In addition, according to the AUC value, the four metabolites and p-tau showed the same power in discriminating AD from iNPH (Figs. 2, 5). Since p-tau is expected to be a good surrogate marker for AD progression [63], the four metabolites indicated here could also be additional surrogate markers. Moreover, metabolic alterations are reportedly seen at a relatively early stage of AD [6, 12, 62], and the four metabolites could be capable of detecting AD earlier than p-tau may detect it.

To validate these results and speculations, utilizing differential metabolite detection methods with additional larger sample size including healthy persons, patients with AD at an early stage, and/or mild cognitive impairment will reveal further information for AD pathogenesis and early diagnosis. We believe that the integration and combination of such information could contribute to developing new diagnostic markers for AD and to expanding the understanding of AD.

## Conclusions

In this study, we found four metabolites that closely participate in the PPP, glycerolipid metabolism, amino acid metabolism, and lipid raft integration were significantly altered in the CSF of patients with AD compared to the CSF of patients with iNPH. All these biological pathways have been demonstrated to be associated with AD in previous reports. Additionally, the combination of these metabolites could discriminate between AD and iNPH with a power equal to that of p-tau and indicated moderate correlation with p-tau. In future studies, the combination of these and additional metabolites included in the metabolic pathways altered in AD would be useful to classify potential patients with AD earlier and with greater precision.

## Additional file

Additional file 1: The list of metabolites detected in the comprehensive metabolic analysis. Cerebrospinal fluid of patients with Alzheimer’s disease and idiopathic normal pressure hydrocephalus was analyzed with capillary electrophoresis-time-of-flight mass spectrometry and detected metabolites were listed. Values are indicated as μM. ND, not detected. (XLSX 135 kb)

## Abbreviations

2-HB: 2-hydroxybutyrate
AD: Alzheimer’s disease
AUC: Area under the ROC curve
CE-ESI-MS: Capillary electrophoresis-electrospray ionization-mass spectrometry
CE-MS: Capillary electrophoresis-mass spectrometry
CE-TOFMS: Capillary electrophoresis-time-of-flight-mass spectrometry
CSF: Cerebrospinal fluid
HPLC: High performance liquid chromatography
iNPH: Idiopathic normal pressure hydrocephalus
Neu5Ac: N-acetylneuraminate
PSS: Phosphatidylserine synthase
